# Weaker Braking Force, A New Marker of Worse Gait Stability in Alzheimer Disease

**DOI:** 10.3389/fnagi.2020.554168

**Published:** 2020-09-11

**Authors:** Qianqian Cheng, Mengxuan Wu, Yuemin Wu, Yaoyao Hu, William Robert Kwapong, Xiang Shi, Yinying Fan, Xin Yu, Jincai He, Zhen Wang

**Affiliations:** ^1^School of Mental Health, Wenzhou Medical University, Wenzhou, China; ^2^Department of Neurology, The First Affiliated Hospital of Wenzhou Medical University, Wenzhou, China; ^3^Department of Neurology, West China Hospital, Chengdu, China; ^4^Wenzhou Yining Geriatric Hospital, Wenzhou, China

**Keywords:** braking force, gait stability, gait variability, fall risk, Alzheimer disease

## Abstract

**Background:** Braking force is a gait marker associated with gait stability. This study aimed to determine the alteration of braking force and its correlation with gait stability in Alzheimer disease (AD).

**Methods:** A total of 32 AD patients and 32 healthy controls (HCs) were enrolled in this study. Gait parameters (braking force, gait variability, and fall risk) in the walking tests of Free walk, Barrier, and Count backward were measured by JiBuEn^®^ gait analysis system. Gait variability was calculated by the coefficient of variation (COV) of stride time, stance time, and swing time.

**Results:** The braking force of AD was significantly weaker than HCs in three walking tests (*P* < 0.001, *P* < 0.001, *P* = 0.007). Gait variability of AD showed significant elevation than HCs in the walking of Count backward (COV_stride_: *P* = 0.013; COV_swing_: *P* = 0.006). Fall risk of AD was significantly higher than HCs in three walking tests (*P* = 0.001, *P* = 0.001, *P* = 0.001). Braking force was negatively associated with fall risks in three walking tests (*P* < 0.001, *P* < 0.001, *P* < 0.001). There were significant negative correlations between braking force and gait variability in the walking of Free walk (COV_stride_: *P* = 0.018; COV_swing_: *P* = 0.013) and Barrier (COV_stride_: *P* = 0.002; COV_swing_: *P* = 0.001), but not Count backward (COV_stride_: *P* = 0.888; COV_swing_: *P* = 0.555).

**Conclusion:** Braking force was weaker in AD compared to HCs, reflecting the worse gait stability of AD. Our study suggests that weakening of braking force may be a new gait marker to indicate cognitive and motor impairment and predict fall risk in AD.

## Introduction

Alzheimer disease (AD) is a neurodegenerative disease characterized by cognitive impairment such as recall, orientation, calculation, attention, and execution, resulting in the decline of life quality, disability, and mortality (Tsai et al., 2019). AD is a chronic and progressive disease with a clinical duration of 8–10 years (Masters et al., 2015). The pathological mechanisms of AD were the deposition of β-amyloid (Aβ) plaques and formation of neurofibrillary tangles recognized as unique characteristics of AD (Jack et al., 2018). The increasing incidence of AD has placed a great economic burden on the world and families (Masters et al., 2015; Alzheimer’s Association, 2019). Currently, no therapy can reverse the underlying mechanisms of the disease. The management of AD is focused on delaying disease progression and treating comorbidities (Masters et al., 2015).

The gait stability of AD is usually worsened, and fall risk is increased (Sheridan et al., 2003). The motor abilities of AD gradually deteriorate, making patients unable to walk eventually. Impaired motor abilities and fall events complicate the disease, leading to poorer prognosis placing a huge burden on caregivers (Schirinzi et al., 2018a). Therefore, a better comprehension of AD-related changes in gait stability may contribute to the development of methods to assess cognitive and motor abilities and interventions to delay dementia and prevent falls.

Cognitive function plays an important role in normal walking, which is required to receive and analyze environmental information and adjust posture to avoid tripping or falling. The impairment of cognition, especially attention, execution, and working memory, may lead to poor gait performance and fall events. Gait abnormalities are not only concomitant symptoms of AD, but also signs of cognitive decline (Montero-Odasso et al., 2012a).

Studies via structural and functional brain imaging have shown that cognition and motor control shared the same brain regions, particularly in the frontotemporal lobes (Montero-Odasso et al., 2017). The “dual-task paradigm” (walking while performing an attention-required task) has been recognized as the optimal test to study the interaction between cognition and motor control (Pelosin et al., 2016). In dual-task, two simultaneous tasks interfere with each other, competing for cortical resources (Montero-Odasso et al., 2012a), thus making it more sensitive to detect the impairment of cognition and motor control, which has been demonstrated in some neurological disorders. With the addition of cognitive tasks, gait abnormalities were more pronounced in patients with AD (Sheridan et al., 2003), Parkinson disease (PD; Pelosin et al., 2016), and multiple sclerosis (Liparoti et al., 2019) than that in controls.

Braking force, a gait marker associated with gait stability, is defined as an active force that reverses the fall of the center of mass (COM), before the swing leg touches the ground during the single-support phase (Chastan et al., 2009).

The goal of postural control while walking is to maintain the COM (a point equivalent of the total body mass in the overall reference system) within the posture basis to maintain balance. When an individual raises his/her leg forward (the raised leg is called swing leg), the COM falls vertically because of gravity. During the single-support phase, the fall of the COM needs to be halted by braking force before the swing leg touches the ground to maintain the COM within the posture basis. For patients with balance disorders, such as PD patients without medication, braking force is absent, and the fall of the COM is halted passively by the touch to the ground of the swing leg (Chastan et al., 2010; Maillot et al., 2014).

Braking force has become an important parameter in the field of biomechanics. The altered braking mechanism could indicate gait instability and falls in the elderly (Maillot et al., 2014), PD, progressive supranuclear palsy (PSP; Chastan et al., 2009), and peripheral neuropathy (Meier et al., 2001). However, the alteration of braking force and its correlation with gait stability in AD have not been studied.

Gait variability is an established marker of gait stability to predict fall events (Montero-Odasso et al., 2012b). It is commonly used to evaluate the gait performance of patients with cognitive impairment in “dual-task paradigm” (Montero-Odasso et al., 2012a). Patients with AD and behavioral variant of frontotemporal dementia (FTD) showed higher gait variability manifested as worse stability parameters in single-task, and the stability parameters further deteriorated in dual-tasks (Rucco et al., 2017).

To sum up, we hypothesized that the braking force of AD is weakened and differs under single- and dual-task, which is associated with worse gait stability reflected by higher gait variability and increased fall risk. This study aimed to determine the changes of braking force, gait variability, and fall risk under single- and dual-task walking in AD patients compared to healthy controls (HCs) and also to explore the relationship between them.

## Materials and Methods

### Study Subjects

In this clinical study, 32 AD patients were recruited from the Memory Clinic of the Neurology Department, the First Affiliated Hospital of Wenzhou Medical University, and 32 HCs were recruited from the physical examination center of the outpatient department. The demographic data such as gender, age, educational level, height, weight, and previous medical history and medication history were collected. All participants completed the Mini-Mental State Examination (MMSE) and reached the following criteria as previously reported (cognitive intact: illiteracy >19, primary school >22, middle school, and above >26; Zhang et al., 1999). The assessments of MMSE were carried out by a well-trained neuropsychologist unaware of the performance in gait tests.

The disease durations of all patients were recorded, and magnetic resonance imaging (MRI) data are all available. According to clinical manifestations, combined with the characteristics of MRI and MMSE, all patients met the “probable AD” diagnostic criteria (1984) of the National Institute of Neurological Speech Disorders and Stroke (Mckhann et al., 1984). The exclusion criteria were as follows: (1) walking requiring assistance or need auxiliary equipment, such as crutches or four-wheel walking aid; (2) diseases of the lower limbs, including muscle atrophy of the lower limbs, knee replacements, hip replacements, or a history of leg fractures within a year; (3) previous history of stroke or other neurological diseases, including PD, multiple sclerosis, myasthenia gravis, cerebellar disease, myelopathy, etc.; (4) have severe mental illness (major depression, bipolar disorder, schizophrenia, alcohol abuse, drug addiction); (5) severely impaired cognitive function or unable to understand and complete the three prescribed walking tests; and (6) unwilling to sign the informed consent.

HCs were free from the above exclusion criteria and other chronic diseases that need long-term medication, such as hypertension and diabetes. HCs were also required to attain the normal range in MMSE mentioned above and be free from cognitive impairment.

This study was approved by the ethics committee of the First Affiliated Hospital of Wenzhou Medical University. Participants signed the informed consent in the presence of a neurological physician before enrollment. Guardian or family members could sign informed consent on behalf of the AD patient when their cognitive function is insufficient to understand the content of the protocol.

### Gait Analysis

Gait performance under single- and dual-task was assessed by an electronic walkway system (JiBuEn^®^ gait analysis system developed and produced by Hangzhou Zhihui Health Management Co., Limited; Xie et al., 2019). In the presence of professional doctors, 32 AD patients and 32 HCs completed all walking test tasks, including a single-task walking Free walk and two dual-task walking Barrier and Count backward. With the help of doctors, all participants wore the gait analysis equipment (a pair of shoes for gait detection and related sensor transmission modules, which were worn on the waist, left thigh, right thigh, left calf, and right calf of the participants). All walking tests were conducted in a quiet environment with a clean floor and a professional escort. We put signs on both ends of the field, 10 m apart. In Free walk, participants were allowed to walk at a comfortable pace without any outside intervention. In Barrier, participants were asked to walk through two obstacles 30 cm apart on the walking path, without giving any hint during the walking. In Count backward, participants were asked to Count backward from 100 while walking, even if the participants’ count performance was wrong.

Stride time was measured as the sum of stance time and swing time (Darweesh et al., 2019). Stride time variability was determined as the coefficient of variation (COV) of stride time and was represented as COV_stride_. Likewise, stance time variability and swing time variability were the COV of stance time and swing time and were represented as COV_stance_ and COV_swing_, respectively (Boripuntakul et al., 2014). The normal range of COV_stride_, COV_stance_, and COV_swing_ were 0% to 10%. The value of braking force was defined as the ratio of the force of the heel strike to the theoretical extremum, and it is no more than 1 (normal range is 0.65–1). Fall risk was calculated by a formula developed by the system according to data collected (Verghese et al., 2009; Taylor et al., 2013), and its normal range is 0% to 15%.

All parameters obtained from the three walking tests were calculated and exported automatically according to the motion signals collected and transmitted by sensors, and the data were stored on a local hard disk.

### Statistical Analysis

Continuous variables were described in terms of mean ± standard deviation (SD) or median [interquartile range (IQR)], depending on whether the data are a normal distribution or not. The normality of data was verified by the Kolmogorov–Smirnov test. Categorical variables were shown in terms of numerical value (percentage). The Student *t*-test was used to compare normal distribution variables. Asymmetrically distributed variables were compared with the Mann–Whitney test. Categorical variables were compared using the *χ*^2^ test. Pearson correlation test was performed for bivariate correlations. Statistical differences of all variables were represented by *P* values, and low-probability events were defined as *P* ≤ 0.05, which was significant. SPSS software (version 22.0 for Windows) was used for the statistical analyses.

## Results

### Demographic and Clinical Characteristics

The demographic and clinical characteristics of the patients with AD and HCs were summarized in Table 1. There was no difference in age and body mass index (BMI) between the two groups (*P* > 0.05). The ratio of female with AD was higher (*P* = 0.044) and the MMSE score of AD was significantly lower (*P* < 0.001) than that of HCs, respectively. The medication history of AD is provided in Supplementary Table S1. The medications taken by patients included cholinesterase inhibitor (ChEI), memantine, selective serotonin reuptake inhibitor (SSRI), and antipsychotics. The specific use ratio and dose are listed in Supplementary Table S1.

**Table 1:** Demographic and clinical characteristics of the samples under study.

	AD (*n* = 32)	HCs (*n* = 32)	*P*
Demographic characteristics		
Sex, female (%)	22 (68.8)	14 (43.8)	0.044
Age, mean ± SD (year) BMI, mean ± SD (kg/m^2^)	67.1 ± 7.4 23.3 ± 2.8	63.6 ± 9.0 23.6 ± 3.2	0.081 0.964
Education levels			0.575
Illiteracy (%)	8 (25.0)	5 (16.1)	0.384
Primary school (%)	10 (31.3)	13 (41.9)	0.378
Middle school and above (%)	14 (43.8)	13 (41.9)	0.884
Disease duration, mean ± SD (year)	3.6 ± 2.1	-	
MMSE score, median (IQR)	14.5 (13.0)	28.0 (2.0)	<0.001

### Gait Parameters

**Figure 1:**
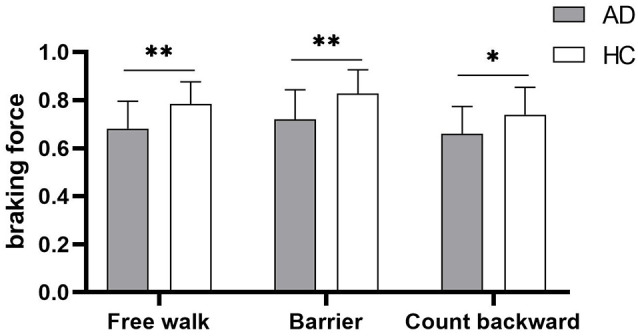
The comparison of braking force between patients with Alzheimer disease (AD) and healthy controls (HCs) in the walking of Free walk, Barrier, and Count backward. *P < 0.05, **P < 0.001.

**Figure 2:**
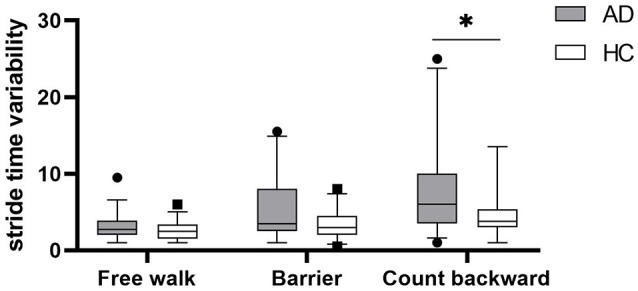
The comparison of stride time variability between patients with AD and HCs in the walking of Free walk, Barrier, and Count backward. *P < 0.05.

**Figure 3:**
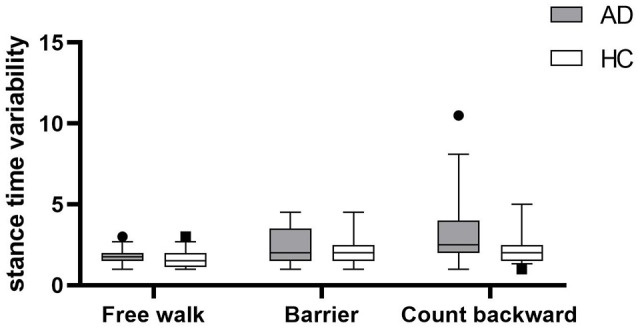
The comparison of stance time variability between patients with AD and HCs in the walking of Free walk, Barrier, and Count backward.

**Figure 4:**
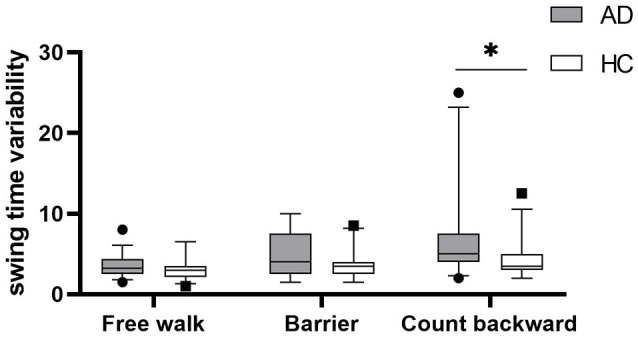
The comparison of swing time variability between patients with AD and HCs in the walking of Free walk, Barrier, and Count backward. *P < 0.05.

Gait parameters are listed in Table 2. COV_stride_ and COV_swing_ showed no difference in the walking of Free walk and Barrier, but significant difference was seen in the walking of Count backward (COV_stride_: *P* = 0.013; COV_swing_: *P* = 0.006) between patients with AD and HCs. COV_stance_ showed no difference in all walking tests. Braking force (*P* < 0.001, *P* < 0.001, *P* = 0.007) and Fall risk (*P* = 0.001, *P* = 0.001, *P* = 0.001) showed a significant difference in all walking tests between patients with AD and HCs, respectively. The braking force of patients with AD and HCs in three tasks is illustrated in Figure 1. The COV_stride_, COV_stance_, and COV_swing_ of patients with AD and HCs in three tasks are illustrated in Figures 2–4. Fall risk was not shown.

**Table 2:** Gait parameters of the samples under study.

	AD (*n* = 32)	HCs (*n* = 32)	*P*
**Stride time variability (%), median (IQR)**			
Free walk	2.5 (1.6)	2.5 (2.0)	0.364
Barrier	3.5 (5.1)	3.0 (2.5)	0.119
Count backward*	5.8 (6.3)	4.0 (2.5)	0.013
**Stance time variability (%), median (IQR)**			
Free walk	1.5 (0.5)	1.5 (1.0)	0.591
Barrier	2.3 (1.6)	2.0 (1.0)	0.240
Count backward	2.3 (1.3)	2.0 (1.0)	0.071
**Swing time variability (%), median (IQR)**			
Free walk	3.0 (1.8)	3.0 (1.5)	0.555
Barrier	4.3 (3.9)	3.5 (1.5)	0.070
Count backward*	5.0 (3.5)	3.5 (2.0)	0.006
**Braking force, mean ± SD**			
Free walk*	0.69 ± 0.11	0.79 ± 0.09	<0.001
Barrier*	0.72 ± 0.12	0.83 ± 0.10	<0.001
Count backward*	0.66 ± 0.11	0.74 ± 0.11	0.007
**Fall risk (%), median (IQR)**			
Free walk*	4.3 (3.4)	2.8 (1.6)	0.001
Barrier*	4.6 (4.0)	2.3 (1.6)	0.001
Count backward*	6.2 (4.4)	3.6 (3.8)	0.001

*P < 0.05 between patients with AD and HCs.

The results of Pearson correlations between gait variability, braking force, and fall risks are shown in Table 3. There were significant negative correlations between braking force and gait variability in Free walk (COV_stride_: *P* = 0.018; COV_swing_: *P* = 0.013; Supplementary Figures S1A,B) and Barrier (COV_stride_: *P* = 0.002; COV_swing_: *P* = 0.001; Supplementary Figures S2A,B), but not Count backward (COV_stride_: *P* = 0.888; COV_swing_: *P* = 0.555; Supplementary Figures S3A,B). There were significant positive correlations between gait variability and fall risk in the walking of Barrier (COV_stride_: *P* = 0.001; COV_swing_: *P* = 0.004) and Count backward (COV_stride_: *P* = 0.024; COV_swing_: *P* = 0.004), but not Free walk (COV _stride_: *P* = 0.587; COV_swing_: *P* = 0.096). However, the braking force was always negatively associated with fall risks in all walking tests (*P* < 0.001, *P* < 0.001, *P* < 0.001), respectively.

**Table 3:** Pearson correlation between gait parameters and braking force/fall risk.

Variables	Braking force		Fall risk	
	*r*	*p*	*r*	*p*
**Free walk**				
Stride time variability	−0.296	0.018	0.069	0.587
Swing time variability	−0.308	0.013	0.210	0.096
Braking force	-	-	-0.533	<0.001
**Barrier**				
Stride time variability	−0.388	0.002	0.426	0.001
Swing time variability	−0.413	0.001	0.365	0.004
Braking force	-	-	-0.515	<0.001
**Count backwards**		
Stride time variability	0.018	0.888	0.285	0.024
Swing time variability	−0.076	0.555	0.358	0.004
Braking force	-	-	-0.471	<0.001

## Discussion

The result of our present study demonstrated that braking force was reduced and fall risk was increased, respectively, in AD when compared to HCs under single- and dual-task walking, but gait variability of AD was significantly higher than HCs only under dual-task walking. Braking force was correlated with fall risk in three walking tests, but correlated with gait variability only in the walking tests without much cognitive distraction.

The fall risk of AD was significantly higher than that of HCs, which is consistent with previous researches (Sheridan et al., 2003; Montero-Odasso et al., 2012a). Braking force was related to fall risk, indicating that braking force could reflect the gait stability of AD to some extent.

The neural mechanism of braking force contributing to gait stability has not been fully understood (Chastan et al., 2009). The research on the correlations between braking force and the structure and function of the brain was sparse. A study on healthy individuals showed that the right prefrontal lobe was activated in the braking process, especially in the inferior frontal gyrus and the preliminary motor cortex (Wang et al., 2009). Besides, the degeneration of the hippocampus would impair visual, vestibular, and proprioceptive perception, resulting in the incomplete reception of the environmental information necessary to maintain normal walking (Annweiler et al., 2012). During the braking process, the brain ought to collect visual information and control muscle locomotion, thus maintaining the proper magnitude and direction of the braking force (Meier et al., 2001). It was reported that the disruption of visual and somatosensory inputs would decrease braking force in healthy adults (Chastan et al., 2010). Therefore, the atrophy of the hippocampus and cortex in AD pathogenesis would weaken the braking force. Moreover, midbrain atrophy was observed in PD and PSP patients with impaired braking force (Chastan et al., 2009, 2010). It remains to be explored what the role of the midbrain is in braking force of AD.

It is worth noting that AD, PD, and PSP are all neurodegenerative diseases, of which cognitive and motor impairments constitute the most common manifestations (Schirinzi et al., 2020). A reduction of Aβ_42_ in cerebrospinal fluid (CSF) was observed in AD, PD, and PSP, which is typically associated with a higher load of Aβ_42_ accumulation in the brain (Blennow et al., 2016; Schirinzi et al., 2018b), thus disrupting neurotransmission and synaptic plasticity and triggering neurodegeneration (Martorana et al., 2015). The concentration of Aβ_42_ in CSF of AD and PSP was inversely proportional to the severity of motor impairment (as reflected in the scores of motor abilities; Schirinzi et al., 2018a,b). Among PD patients, the diffuse malignant subtype with more severe cognitive and motor impairment showed AD-like CSF profile with lower levels of Aβ_42_, when compared to other subtypes. This result is consistent with those identified in postmortem studies in which the diffuse malignant subtype had more Aβ plaques and cortical degeneration (Fereshtehnejad et al., 2017). These findings suggest that the amyloidosis might be associated with motor impairment in neurodegeneration.

Furthermore, Aβ_42_ can deposit in cholinergic nuclei, interfering with cholinergic system, which is susceptible to Aβ_42_-related degeneration (Schirinzi et al., 2018b). Cholinergic system plays a vital role in motor control. Cholinergic transmission is involved in attention and executive function, and its disruption can lead to impaired attentional ability and increased fall risk. It has been suggested that cholinergic transmission was disrupted in AD (Schirinzi et al., 2018a), PD and PSP (Gilman et al., 2010), and cholinergic dysfunction was correlated with motor impairment in AD (Schirinzi et al., 2018a). The aforementioned studies on AD, PD, and PSP, in which braking force was proven weakened, suggest a possible underlying mechanism that the amyloidosis of AD pathogenesis is likely to impair motor control and weaken braking force by interfering with cholinergic transmission.

Central cholinergic activity showed progressive attenuation among old nonfallers, old fallers, and PD patients, and it was negatively correlated with dual-task cost. Cholinergic dysfunction may disrupt attention distribution, which is prominent in dual-task, resulting in impaired motor control and increased risks of falling (Pelosin et al., 2016). In dual-tasks, attention is assigned to cognitive tasks, so that gait stability is not well maintained. Cholinergic dysfunction of AD heightened dual-task cost, and a more difficult cognitive task with a larger need for cortical resources produced greater dual-task cost. Therefore, the differences of COV_stride_ and COV_swing_ between patients with AD and HCs enlarged as the walking tasks became more complicated, and significant differences were shown only in dual-task walking while counting backward. It turned out that gait variability was more sensitive to dual-task in reflecting cognitive function, which was consistent with previous researches (Montero-Odasso et al., 2012a,b). However, the braking force did not show this trend in our results. Further studies are required to verify the effect of cholinergic activity on braking force and gait variability.

We continued to explore the correlation between braking force and gait variability. This is the first time to list stride time and its components stance time and swing time simultaneously to compare the correlations among them. COV_swing_ showed the same trends as COV_stride_ in different groups and conditions, whereas COV_stance_ did not. It could be inferred that COV_stride_ was mainly influenced by COV_swing_. The swing phase of gait was supposed to play a vital role in gait stability. Braking force functions in the single-support phase, which is the swing phase, to maintain gait stability. This just proved the correlation between braking force and gait variability. The results of Pearson correlation test shown in Table 3 and Supplementary Figures S1–S3A,B also verified that braking force was correlated with gait variability, but only in Free walk and Barrier, not in Count backward. Cognitive requirements significantly increased gait variability but slightly reduced braking force.

A kinematics study in patients with AD and FTD can shed some light on the explanation of the results of braking force. It was found that the range of motion (RoM, reflecting the magnitude of joint excursion) was reduced in these two groups, respectively, when compared to HCs in single- and dual-tasks (Rucco et al., 2017). From the biomechanical analysis, in single-task, the RoM of AD was only impaired in the swing phase, which is a critical period for joint motion to maintain dynamic stability (Rucco et al., 2017), whereas with the addition of cognitive task, the RoM of AD was impaired in both stance and swing phase (Rucco et al., 2017). Because braking force does not work in the stance phase (Maillot et al., 2014), it is elucidated that the effect of the cognitive task on gait has little correlation with braking force. As for the performance of FTD, the impairment of RoM was worse than that of AD, but it did not deteriorate with the addition of cognitive tasks (Rucco et al., 2017), similar to the presentation of braking force. The pathogenesis of FTD is the atrophy of the frontotemporal lobes (Rucco et al., 2017). As mentioned previously, the motor control area is mainly in the frontotemporal lobes (Montero-Odasso et al., 2017), and the area associated with braking force is the prefrontal cortex (Wang et al., 2009). It might be inferred that weaker braking force may be involved only in the lesion of frontotemporal lobes responsible for motor control in the course of AD. This inference needs further investigations by biomechanical researches.

In addition, it is necessary to mention the effect of drugs on gait. ChEI and memantine have been developed for the treatment of AD (Sharma, 2019). ChEI and memantine treatment could not only decrease gait variability in AD (Montero-Odasso et al., 2009; Beauchet et al., 2013), but also improve balance and stability in PD (Devos et al., 2010; Henderson et al., 2016; Lauretani et al., 2016). It is suggested that the gait abnormalities in AD samples were underestimated because of their use of antidementia drugs. However, a relatively low percentage of patients took SSRI and antipsychotics because of the accompanying emotional and psychotic symptoms. It was reported that SSRI and psychotropic use could increase fall risk (Liu et al., 1998; Leipzig et al., 1999). Drug use, as a covariable of gait, would take a certain effect on the gait performance driven by AD pathogenesis itself. More precise studies are needed to control drug use.

Individuals with cognitive impairment are at high risk of falling, but interventions that took effect for individuals with normal cognition may not work well for them. It was inferred that the mechanisms of falling may be different in them (Montero-Odasso et al., 2012a). Measuring cognitive-related gait markers, such as braking force and gait variability, could better reflect the disease severity and fall risk of AD, providing reference for nursing and rehabilitation.

We would like to acknowledge that this cross-sectional study was preliminary and presented some limitations. The confounding of covariates and the lack of longitudinal observation make us conservative about the revelation of weaker braking force on gait instability in AD. Further confirmatory studies are required to adjust potential covariates including the drug utilization and conduct follow-up over the duration. Besides, the small sample limited the analysis of multiple cognitive domains. It might make sense to clarify the relationship between braking force and some cognitive domains, particularly visual–spatial capacity and executive function. Possibly, it may explain more about the decline of braking force in AD to analyze its association with the alteration in cortex and subcortex by functional imaging, as well as muscle joint movement by biomechanical studies. Another important limitation to our study is the poor characterization of the AD patients; we did not perform lumbar puncture or positron emission tomography neuroimaging to detect the presence of Aβ pathology in our AD patients. Moreover, even though the screening of cognitive examination with MMSE was done in each subject, it cannot be ruled out with confidence that among the HCs are Aβ-positive people. Further efforts are necessary, aiming at optimizing the inclusion criteria and research methodology, to enhance the reliability of the research results.

Despite these limitations, there are some strengths in our study. We pioneered the study of braking force in dementia and explored its correlations with common gait markers. Our data suggest that weaker braking force is related to worse gait stability of AD, and patients with AD may benefit from gait examination, serving as a speculative foundation for practical methodologies.

## Conclusion

Our study first found that the weaker braking force was a sign of worse gait stability in AD. It was negatively correlated with fall risk and correlated with gait variability under the condition without much cognitive distraction. Braking force is expected to be a novel gait marker to estimate fall risk without the addition of cognitive tasks. Further prospective researches are deserved to investigate its correlation with cognition, motor control, and gait variability.

## Data Availability Statement

The raw data supporting the conclusions of this article will be made available by the authors, without undue reservation.

## Ethics Statement

The studies involving human participants were reviewed and approved by the ethics committee of the first affiliated hospital of Wenzhou Medical University. The patients/participants provided their written informed consent to participate in this study.

## Author Contributions

ZW designed the study. QC, MW, YW, YH, YF and XS all participated in organizing subjects and data acquisition. QC did the statistical analysis, then interpreted the data and made the corresponding tables and figures. QC and MW drafted the manuscript. WK revised the manuscript. ZW, JH and XY supervised the study. All contributing researchers are listed here. All authors contributed to the article and approved the submitted version.

## Conflict of Interest

The authors declare that the research was conducted in the absence of any commercial or financial relationships that could be construed as a potential conflict of interest.

## References

[B1] Alzheimer’s Association (2019). 2019 Alzheimer’s disease facts and figures. Alzheimers Dement. 15, 321–387. 10.1016/j.jalz.2019.01.010

[B2] Annweiler C. Beauchet O. Celle S. Roche F. Annweiler T. Allali G.. (2012). Contribution of brain imaging to the understanding of gait disorders in Alzheimer’s disease: a systematic review. Am. J. Alzheimers Dis. Other Demen. 27, 371–380. 10.1177/1533317512454710 22930697 PMC11008139

[B3] Beauchet O. Launay C. P. Allali G. Watfa G. Gallouj K. Herrmann F. R.. (2013). Anti-dementia drugs and changes in gait: a pre-post quasi-experimental pilot study. BMC Neurol. 13:184. 10.1186/1471-2377-13-184 24261605 PMC3898226

[B4] Blennow K. Biscetti L. Eusebi P. Parnetti L. (2016). Cerebrospinal fluid biomarkers in Alzheimer’s and Parkinson’s diseases-From pathophysiology to clinical practice. Mov. Disord. 31, 836–847. 10.1002/mds.26656 27145480

[B5] BoripuntakulS.LordS. R.BrodieM. A. D.SmithS. T.MethapataraP.WongpakaranN.. (2014). Spatial variability during gait initiation while dual tasking is increased in individuals with mild cognitive impairment. J. Nutr. Health Aging 18, 307–312. 10.1007/s12603-013-0390-324626760

[B6] Chastan N. Westby G. W. M. du Montcel S. T. Do M. C. Chong R. K. Agid Y.. (2010). Influence of sensory inputs and motor demands on the control of the centre of mass velocity during gait initiation in humans. Neurosci. Lett. 469, 400–404. 10.1016/j.neulet.2009.12.038 20026383

[B7] Chastan N. Do M. C. Bonneville F. Torny F. Bloch F. Westby G. W. M.. (2009). Gait and balance disorders in Parkinson’s disease: impaired active braking of the fall of centre of gravity. Mov. Disord. 24, 188–195. 10.1002/mds.22269 18973252

[B8] Darweesh S. K. L. Licher S. Wolters F. J. Koudstaal P. J. Ikram M. K. Ikram M. A.. (2019). Quantitative gait, cognitive decline and incident dementia: the rotterdam study. Alzheimers Dement. 15, 1264–1273. 10.1016/j.jalz.2019.03.013 31515066

[B9] Devos D. Bordet R. Defebvre L. (2010). Pharmacological hypotheses and therapeutic strategies for gait disorders in Parkinson’s disease. Rev. Neurol. 166, 168–177. 10.1016/j.neurol.2009.07.017 19811797

[B10] Fereshtehnejad S.-M. Zeighami Y. Dagher A. Postuma R. B. (2017). Clinical criteria for subtyping Parkinson’s disease: biomarkers and longitudinal progression. Brain 140, 1959–1976. 10.1093/brain/awx118 28549077

[B11] Gilman S. Koeppe R. A. Nan B. Wang C.-N. Wang X. Junck L.. (2010). Cerebral cortical and subcortical cholinergic deficits in parkinsonian syndromes. Neurology 74, 1416–1423. 10.1212/wnl.0b013e3181dc1a55 20439843 PMC2871002

[B12] Henderson E. J. Lord S. R. Brodie M. A. Gaunt D. M. Lawrence A. D. Close J. C. T.. (2016). Rivastigmine for gait stability in patients with Parkinson’s disease (ReSPonD): a randomised, double-blind, placebo-controlled, phase 2 trial. Lancet Neurol. 15, 249–258. 10.1016/S1474-4422(15)00389-0 26795874

[B13] Jack C. R. Jr. Bennett D. A. Blennow K. Carrillo M. C. Dunn B. Haeberlein S. B.. (2018). NIA-AA research framework: toward a biological definition of Alzheimer’s disease. Alzheimers Dement. 14, 535–562. 10.1016/j.jalz.2018.02.018 29653606 PMC5958625

[B14] Lauretani F. Galuppo L. Costantino C. Ticinesi A. Ceda G. Ruffini L.. (2016). Parkinson’s disease (PD) with dementia and falls is improved by AChEI? a preliminary study report. Aging Clin. Exp. Res. 28, 551–555. 10.1007/s40520-015-0437-x 26294137

[B15] Leipzig R. M. Cumming R. G. Tinetti M. E. (1999). Drugs and falls in older people: a systematic review and meta-analysis: I. Psychotropic drugs. J. Am. Geriatr. Soc. 47, 30–39. 10.1111/j.1532-5415.1999.tb01898.x 9920227

[B16] Liu B. Anderson G. Mittmann N. To T. Axcell T. Shear N. (1998). Use of selective serotonin-reuptake inhibitors or tricyclic antidepressants and risk of hip fractures in elderly people. Lancet 351, 1303–1307. 10.1016/s0140-6736(97)09528-7 9643791

[B17] Liparoti M. Corte M. D. Rucco R. Sorrentino P. Sparaco M. Capuano R.. (2019). Gait abnormalities in minimally disabled people with multiple sclerosis: a 3D-motion analysis study. Mult. Scler. Relat. Disord. 29, 100–107. 10.1016/j.msard.2019.01.028 30703704

[B18] Maillot P. Perrot A. Hartley A. Do M.-C. (2014). The braking force in walking: age-related differences and improvement in older adults with exergame training. J. Aging Phys. Act. 22, 518–526. 10.1123/japa.2013-0001 24231655

[B19] Martorana A. Di Lorenzo F. Belli L. Sancesario G. Toniolo S. Sallustio F.. (2015). Cerebrospinal fluid Aβ42 levels: when physiological become pathological state. CNS Neurosci. Ther. 21, 921–925. 10.1111/cns.12476 26555572 PMC6493161

[B20] Masters C. L. Bateman R. Blennow K. Rowe C. C. Sperling R. A. Cummings J. L. (2015). Alzheimer’s disease. Nat. Rev. Dis. Primers 1:15056. 10.1038/nrdp.2015.56 27188934

[B21] Mckhann G. Drachman D. Folstein M. Katzman R. Price D. Stadlan E. M. (1984). Clinical diagnosis of Alzheimer’s disease: report of the NINCDS-ADRDA work group under the auspices of department of health and human services task force on Alzheimer’s disease. Neurology 34, 939–944. 10.1212/wnl.34.7.939 6610841

[B22] Meier M. R. Desrosiers J. Bourassa P. Blaszczyk J. (2001). Effect of type II diabetic peripheral neuropathy on gait termination in the elderly. Diabetologia 44, 585–592. 10.1007/s001250051664 11380076

[B23] Montero-Odasso M. Verghese J. Beauchet O. Hausdorff J. M. (2012a). Gait and cognition: a complementary approach to understanding brain function and the risk of falling. J. Am. Geriatr. Soc. 60, 2127–2136. 10.1111/j.1532-5415.2012.04209.x 23110433 PMC3498517

[B24] Montero-Odasso M. Muir S. W. Speechley M. (2012b). Dual-task complexity affects gait in people with mild cognitive impairment: the interplay between gait variability, dual tasking and risk of falls. Arch. Phys. Med. Rehabil. 93, 293–299. 10.1016/j.apmr.2011.08.026 22289240

[B25] Montero-Odasso M. M. Sarquis-Adamson Y. Speechley M. Borrie M. J. Hachinski V. C. Wells J.. (2017). Association of dual-task gait with incident dementia in mild cognitive impairment: results from the gait and brain study. JAMA Neurol. 74, 857–865. 10.1001/jamaneurol.2017.0643 28505243 PMC5710533

[B26] Montero-Odasso M. Wells J. Borrie M. (2009). Can cognitive enhancers reduce the risk of falls in people with dementia? An open-label study with controls. J. Am. Geriatr. Soc. 57, 359–360. 10.1111/j.1532-5415.2009.02085.x 19207156 PMC5017867

[B27] Pelosin E. Ogliastro C. Lagravinese G. Bonassi G. Mirelman A. Hausdorff J. M.. (2016). Attentional control of gait and falls: is cholinergic dysfunction a common substrate in the elderly and Parkinson’s disease? Front. Aging Neurosci. 8:104. 10.3389/fnagi.2016.00104 27242515 PMC4860418

[B28] Rucco R. Agosti V. Jacini F. Sorrentino P. Varriale P. De Stefano M.. (2017). Spatio-temporal and kinematic gait analysis in patients with frontotemporal dementia and Alzheimer’s disease through 3D motion capture. Gait Posture 52, 312–317. 10.1016/j.gaitpost.2016.12.021 28038340

[B29] Sheridan P. L. Solomont J. Kowall N. Hausdorff J. M. (2003). Influence of executive function on locomotor function: divided attention increases gait variability in Alzheimer’s disease. J. Am. Geriatr. Soc. 51, 1633–1637. 10.1046/j.1532-5415.2003.51516.x 14687395

[B30] Schirinzi T. Canevelli M. Suppa A. Bologna M. Marsili L. (2020). The continuum between neurodegeneration, brain plasticity and movement: a critical appraisal. Rev. Neurosci. 20200011. 10.1515/revneuro-2020-0011 32678804

[B31] Schirinzi T. Di Lorenzo F. Sancesario G. M. Di Lazzaro G. Ponzo V. Pisani A.. (2018a). Amyloid-mediated cholinergic dysfunction in motor impairment related to Alzheimer’s disease. J. Alzheimers Dis. 64, 525–532. 10.3233/jad-171166 29914023

[B32] Schirinzi T. Sancesario G. M. Di Lazzaro G. Scalise S. Colona V. L. Imbriani P.. (2018b). Clinical value of CSF amyloid-beta-42 and tau proteins in progressive supranuclear palsy. J. Neural. Transm. 125, 1373–1379. 10.1007/s00702-018-1893-1 29948175

[B33] Sharma K. (2019). Cholinesterase inhibitors as Alzheimer’s therapeutics (Review). Mol. Med. Rep. 20, 1479–1487. 10.3892/mmr.2019.10374 31257471 PMC6625431

[B34] Taylor M. E. Delbaere K. Mikolaizak A. S. Lord S. R. Close C. T. (2013). Gait parameter risk factors for falls under simple and dual task conditions in cognitively impaired older people. Gait Posture 37, 126–130. 10.1016/j.gaitpost.2012.06.024 22832468

[B35] Tsai C.-L. Liang C.-S. Lee J.-T. Su M.-W. Lin C.-C. Chu H.-T.. (2019). Associations between plasma biomarkers and cognition in patients with Alzheimer’s disease and amnestic mild cognitive impairment: a cross-sectional and longitudinal study. J. Clin. Med. 8:1893. 10.3390/jcm8111893 31698867 PMC6912664

[B36] Verghese J. Holtzer R. Lipton R. B. Wang C. (2009). Quantitative gait markers and incident fall risk in older adults. J. Gerontol. A Biol. Sci. Med. Sci. 64, 896–901. 10.1093/gerona/glp033 19349593 PMC2709543

[B37] Wang J. J. Wai Y. Y. Weng Y. H. Ng K. K. Huang Y.-Z. Ying L.. (2009). Functional MRI in the assessment of cortical activation during gait-related imaginary tasks. J. Neural. Transm. 116, 1087–1092. 10.1007/s00702-009-0269-y 19669694

[B38] Xie H. Wang Y. Tao S. Huang S. Zhang C. Lv Z. (2019). Wearable sensor-based daily life walking assessment of gait for distinguishing individuals with amnestic mild cognitive impairment. Front. Aging Neurosci. 11:285. 10.3389/fnagi.2019.00285 31695605 PMC6817674

[B39] Zhang Z. Hong X. Li H. (1999). The mini-mental state examination in the chinese residents population aged 55 years old and over in the urban and rural areas of beijing. Chin. J. Neurol. 3, 3–5.

