# Supplementary material for: Weaker Braking Force, A New Marker of Worse Gait Stability in Alzheimer Disease
Source: Front Aging Neurosci. 2020 Sep 11;12:554168. doi: 10.3389/fnagi.2020.554168 (PMC7516124; doi:10.3389/fnagi.2020.554168)
Supplement: Supplementary file 1 [file Table_1.DOCX]

**Table S1** The medication history of AD.

| Drug | Use ratio (%) | Average dose of last month of gait test (mg/d) |
| --- | --- | --- |
| ChEI (donepezil) | 93.8 | 5.6 |
| Memantine | 62.5 | 10.8 |
| SSRI (sertraline) | 9.4 | 50 |
| Antipsychotics | 18.8 |  |
| olanzapine | 6.3 | 10 |
| risperidone | 6.3 | 1 |
| quetiapine | 3.1 | 75 |
| clozapine | 3.1 | 75 |

ChEI: Cholinesterase Inhibitor, SSRI: Selective Serotonin-Reuptake Inhibitor
