# Supplementary material for: Weaker Braking Force, A New Marker of Worse Gait Stability in Alzheimer Disease
Source: Front Aging Neurosci. 2020 Sep 11;12:554168. doi: 10.3389/fnagi.2020.554168 (PMC7516124; doi:10.3389/fnagi.2020.554168)
Supplement: Supplementary file 7 [file Data_Sheet_6.PDF]

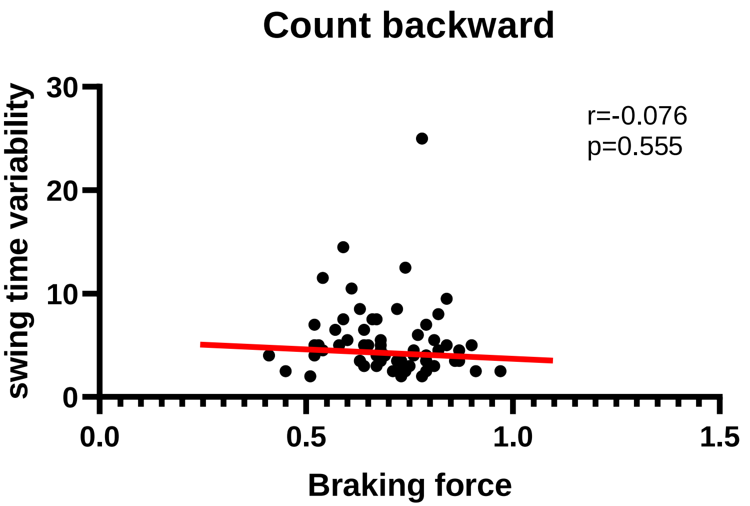

Figure S3B: The correlation of braking force with swing time variability in the walking of Count backward.
